# Supplementary material for: Content-rich biological network constructed by mining PubMed abstracts
Source: BMC Bioinformatics. 2004 Oct 8;5:147. doi: 10.1186/1471-2105-5-147 (PMC528731; doi:10.1186/1471-2105-5-147)
Supplement: Additional File 5 — The original Chilibot query results of the term "long-term potentiation (LTP)" and 22 other terms, limiting the latest references analyzed to the years 1990, 1995, 2000, and 2004. [file 1471-2105-5-147-S5.bz2 › chilibotAdditionalFile5/ltp2004/html/CAMKII_ACTININ.html]

 


 **CAMKII** and **ACTININ** 
  
Found 6 abstracts in PubMed,  **6 abstracts were retrieved and analyzed**.  


---

 Search Google  |
 PDF files only 
|  EDU domain only 

---

**Interactive relationship** (e.g. stimulation, inhibition, etc)

- **CaMKII**  as well as the previously identified NR1 binding partners calmodulin and alpha  **actinin**  bind to the short C terminal portion of the C0 region of NR1.  Ref: 12379661 J Biol Chem, 2002
- We demonstrate by yeast two hybrid and biochemical assays that the intracellular portion of densin 180, the alpha subunit of  **CaMKII**  CaMKIIalpha, and alpha  **actinin**  interact with each other at distinct binding sites and can form a ternary complex stabilized by multiple interactions.  Ref: 11160423 J Neurosci, 2001
- Like Ca calmodulin, autophosphorylated  **CaMKII**  competes with alpha  **actinin**  2 for binding to NR1.  Ref: 12379661 J Biol Chem, 2002
- A distinct domain of alpha  **actinin**  interacts with the kinase domains of both alpha and beta subunits of  **CaMKII** .  Ref: 11160423 J Neurosci, 2001
- We also suggest how recently discovered binding interactions could provide a structural readout mechanism the autophosphorylated state of  **CaMKII**  binds tightly to the NMDAR and forms, through  **CaMKII**   **actinin**  actin 4.1 SAP97 linkages, additional sites for anchoring AMPARs at synapses.  Ref: 11502252 Neuron, 2001
- The results suggest that beta1 integrin is tyrosine phosphorylated and links with actin via alpha  **actinin**  in HBE cells but prevented from linking with actin in MCF 7 cells by phosphorylation at both tyrosine and serine threonine of beta1 integrin which forms a complex with alpha  **actinin**  and  **CaMKII** .  Ref: 11716783 BMC Cell Biol, 2001

**Parallel relationship** (e.g. studied together, co-existance, homology, etc.)

- We conclude that the NR1 C0 region is a key site for recruiting  **CaMKII**  to the postsynaptic site, where it may act in concert with calmodulin to modulate the stimulatory role of alpha  **actinin**  interaction with the NMDA receptor.  Ref: 12379661 J Biol Chem, 2002
- Here we report the identification of the alpha subunit of calcium calmodulin dependent protein kinase II  **CaMKII**  and alpha  **actinin**  4 as potential binding partners for the densin 180 intracellular segment.  Ref: 11160423 J Neurosci, 2001
